# A new perspective on the biguanide, metformin therapy in type 2 diabetes and lactic acidosis

**DOI:** 10.1111/jdi.13090

**Published:** 2019-07-15

**Authors:** Nigishi Hotta

**Affiliations:** ^1^ Department of Internal Medicine Chubu Rosai Hospital Japan Organization of Occupational Health and Safety Nagoya Japan

## Abstract

Recent topics about metformin mainly focus on clinical studies, briefly mentioning some new aspects on the mechanisms of metformin action.

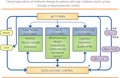

Metformin is the most prescribed oral hypoglycemic agent for the treatment of type 2 diabetes in Western countries^1^. Metformin is now becoming a more popular drug for type 2 diabetes in Japan too.

It is well accepted that the antihypoglycemic actions of metformin include the reduction of gluconeogenesis in the liver and the increase of glucose uptake in skeletal muscle tissue. Recently, some interesting information has emerged that the gut might play an important role in hypoglycemic action by metformin^2^ through microbiota^3^, the gut–brain–liver axis^4^ or the gut–pancreas network.

From the viewpoint of clinical practice, there are many useful reports of clinical trials using metformin alone or in combination therapy with other drugs. In Japan, the recommended frequency of metformin is commonly three times or twice per day. No previous study has observed the effects of the dosing frequency of metformin on the relationship between daily blood glucose profile and plasma concentration of metformin in patients treated with this drug. A recent study^5^ clearly showed no difference in the daily blood glucose profile between the twice‐ and three times‐daily metformin (1,500 mg per day) treatment groups, despite some differences in the daily profile of plasma metformin concentration between these two groups. Thus, these data might provide a means of improving adherence and metabolic target achievement with treatment by metformin, because as either the frequency or number of pills for administration increases, non‐adherence to medication becomes very common^6^. Metformin is usually taken before each meal. This common sense was clearly confirmed in a recent study on the efficacy of preprandial metformin administration on postprandial plasma triglyceride concentration in patients with type 2 diabetes^7^. The authors observed that preprandial metformin administration significantly reduced plasma triglyceride levels during meal testing, as compared with no significant reduction in the group with postprandial metformin administration. However, there were no differences in blood glucose level and plasma insulin concentration between these two groups. From the view of metabolic target achievement in the long term, the reduction of plasma triglyceride levels after meals by metformin can contribute to preventing or slowing the development of related diabetic complications or insulin resistance. Therefore, it indicates that metformin administration is crucial before each meal.

Secondary failure of oral hypoglycemic agents during long‐term medication is a concern for patients and medical staff. A recent report by a Korean group on a prospective, multicenter, observational cohort study with 56 months of median follow‐up time showed that metformin was associated with a lower failure risk, as compared with sulfonylureas and meglitinides, from data on monotherapy in patients with type 2 diabetes^8^. As this kind of report is rare, it is valuable. Lactic acidosis is another serious concern for diabetes patients. When metformin is prescribed for people with kidney or liver disease and in high doses, they might encounter lactic acidosis. Liu *et al*.^9^ reported the first case of the effect of extracorporeal metformin elimination in a woman with a metformin overdose, especially using resin‐based sorbent hemoperfusion. The case was a 42‐year‐old woman who attempted suicide by taking approximately 100 tablets of metformin (500 mg), showing severe lactic acidosis with hyperlactemia (24 mmol/L) and acidemia (pH 7.09.) Regulatory and professional society guidelines suggest that metformin might be an option for patients with mild‐to‐moderate chronic kidney disease. The US Food and Drug Administration does not recommend initiating metformin at estimated glomerular filtration rate <45 mL/min/1.73 m^2^. A recent report of a community‐based cohort study supports cautious prescription of metformin for patients with type 2 diabetes and estimated glomerular filtration rate of ≥30 mL/min/1.73 m^2^ 10. However, we must always consider the patient's condition and differences by race.

The presence of hyperglycemia, hypoglycemia and overweight is a matter of concern with pharmacotherapy in daily practice. However, these anxieties are not always easily resolved. Recently, the following four studies showed a positive outlook. In a randomized trial to compare the effects of metformin, gliclazide and liraglutide, a glucagon‐like peptide‐1 analog, on body composition in patients with both type 2 diabetes and non‐alcoholic fatty liver disease, either of these drug monotherapies for 24 weeks resulted in greater weight loss, reduction in body mass and better blood glucose control, contributing to a favorable hepatic function^11^. In that study, body composition was measured using dual‐energy X‐ray absorptiometry. In contrast, in a similar investigation if people with type 2 diabetes using metformin and alogliptin, a dipeptidyl peptidase‐4 inhibitor monotherapy, for 12 weeks proves that the higher the body mass index, the more metformin reduces bodyweight and alogliptin increases bodyweight^12^. These two studies strongly suggest that metformin is a very useful means for better glycemic control with a reduction in bodyweight in type 2 diabetes patients. In a study using continuous glucose monitoring, it was reported that the combination therapy of low‐dose metformin (750 mg) and linagliptin, a dipeptidyl peptidase‐4 inhibitor, or high‐dose metformin (1,500 mg), can improve post‐breakfast glycemic variability in type 2 diabetes patients with insufficient glycemic control with low‐dose metformin (500–1,000 mg) monotherapy^13^. We learn from that study that continuous glucose monitoring and suitable combination therapy with metformin are very helpful in trying to stabilize glycemic variability. In regard to combination therapy, a study of combination therapy of insulin and sitagliptin, a dipeptidyl peptidase‐4 inhibitor, in type 2 diabetes patients for 24 weeks showed that the addition of sitagliptin to inadequate glycemic control receiving stable insulin therapy with or without metformin led to remarkably good glycemic control compared with placebo^14^. A matter of concern in that study is the frequency of hypoglycemia. Adverse events of hypoglycemia (symptomatic or asymptomatic) were observed in a total of 64 (27.4%) patients taking sitagliptin, and 51 (21.9%) patients taking placebo. There were no significant differences between the two groups. It is always crucial for us to pay attention to hypoglycemia in patients receiving treatment with glucose‐lowering agents, resulting in the development and/or the aggravation of diabetic complications.

The present article on recent topics about metformin mainly focuses on clinical studies, briefly mentioning some aspects of the mechanisms of metformin actions. Although metformin is already expected to have anti‐aging effects and glucose‐lowering effects through the gut microbiome, promising future therapeutic targets of metformin are increasing, including reducing the risk of dementia‐type neurodegenerative disorders and certain cancers. All of these aspects are summarized in Figure 1,^15^ as well as how actions by metformin are related, with or without glucose‐lowering effects.

**Figure 1:**
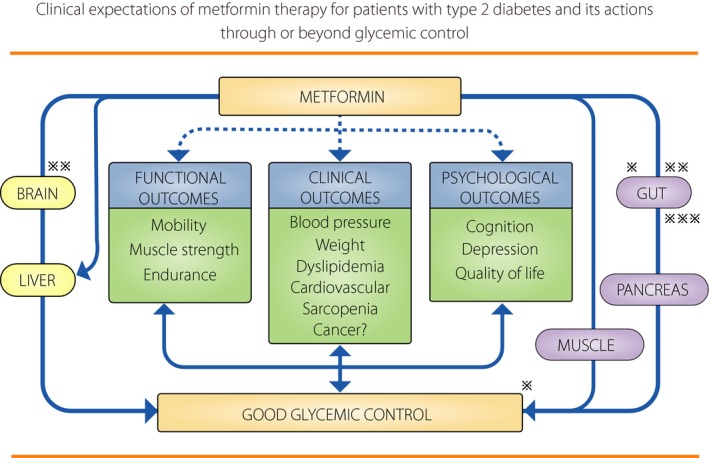
Potential clinical targets for metformin in type 2 diabetes: Dependent or independent of blood glucose control. Through a long history of metformin actions in clinical and experimental studies, we are learning that metformin may have useful actions for the diabetic state, dependent or independent of blood glucose control. However, the exact mechanisms or pathways are not always clear yet in detail. It is well known that most metformin actions in metabolic pathways may be related to adenosine mono‐phosphate kinase (AMPK) activity in the target cells. ※ It is unknown yet how the gut microbiome interacts with the host targets in improving host metabolism including glucose lowering effects^3^). ※※ It is proposed that metformin activates a duodenal AMPK‐dependent neuronal pathway to lower hepatic glucose production and plasma glucose levels in diabetes^4^). ※※※ Metformin impacts on glucose metabolism by including glucagon‐like peptide‐1 (GLP‐1) increment, contributing to increase insulin secretion. [Colour figure can be viewed at wileyonlinelibrary.com]

## Disclosure

The author declares no conflict of interest.
